# OA17 Rheumatoid meningitis - a rare complication of rheumatoid arthritis

**DOI:** 10.1093/rap/rkad070.017

**Published:** 2023-09-27

**Authors:** Asma Ibrahim, Adrian Jones

**Affiliations:** Nottingham University Hospital NHS Trust, Nottingham, United Kingdom; Nottingham University Hospital NHS Trust, Nottingham, United Kingdom

## Abstract

**Introduction:**

A wide spectrum of neurological complications can occur in rheumatoid arthritis (RA) including peripheral neuropathy, mononeuritis multiplex and rheumatoid vasculitis. Cerebrovascular disease is also common. We describe a rare neurological complication of RA.

**Case description:**

The patient was first diagnosed with seropositive RA in 2015 at the age of 68. His rheumatoid factor at presentation was 144 kIU/L and CCP antibodies >340 U/mL. His background include alcohol excess and obstructive sleep apnoea with peripheral neuropathy attributed to alcohol. Nerve conduction studies demonstrated axonal neuropathy. By 2016, he had achieved good disease control with combination methotrexate and sulfasalazine.

In 2018, he was admitted to stroke services with right arm weakness and focal neurological deficit on examination. An urgent CT head showed possible right parietal extra dural haemmorhage. His CRP was <5, ESR 24 with a normal ANA. Borrelia, syphilis and HIV screening were normal. Anti-glutamic acid decarboxylase (GAD), anti-amphiphysin, anti-Hu, anti-Yo and anti-Ri antibodies were all negative. Lumbar puncture revealed a normal glucose and red blood cells count but the CSF protein was raised at 851 mg/L with a lymphocyte count of 9/ul. Gram stain, culture, viral PCR for HSV, VZV, enterovirus, parechovirus and adenovirus were normal and CSF cytology demonstrated a lymphocyte pleocytosis. Subsequent contrast MRI head scan showed leptomeningeal enhancement with gadolinium contrast.

MRI of the cervical spine and brachial plexus was normal and an EMG/NCS of the upper limb demonstrated an incidental ulnar nerve compression at the elbow. Following neurology input, the leptomeningeal enhancement was thought to represent rheumatoid meningitis (RM). He was treated with 1 mg/kg prednisolone and rituximab with prompt and complete recovery. He remains well from both the perspective of his RA and RM.

**Discussion:**

RM is a rare form of aseptic meningitis. Aseptic meningitis is commonly defined as clinical and CSF evidence of meningeal inflammation in the absence of an infectious aetiology. The vast differential includes malignancies, drug-induced and autoimmune diseases, for example lupus and sarcoidosis. The diagnosis of RM requires a high index of suspicion as well as exclusion of other causes. Historically RM was diagnosed at post-mortem but MRI scanning means pre-mortem diagnosis is possible. It is considered rare but serious manifestation of RA. Case reports suggest RM usually occurs in those with long standing seropositive RA but RM has been described in the early stages of RA and even as a presenting feature. Peripheral disease activity does not correlate with occurrence of RM. The clinical presentations vary depending on the brain structure involved and can include motor or sensory deficits, cognitive disturbance and seizures. It may mimic many other conditions. Radiological findings will not distinguish RM from other causes of aseptic meningitis. CSF finding of lymphocytosis and rheumatoid factor (if available) may help distinguish RM from other causes. Meningeal biopsy can confirm the diagnosis.

Due to the rarity of RM, there are no current guidelines on treatment. Historically, corticosteroids are the main induction agent. There are case reports highlighting possible success from the addition of cyclophosphamide and rituximab. Case series also highlight a significant mortality rate in excess of 60% even despite treatment. This may represent publication and historical bias.

**Key learning points:**

Rheumatoid meningitis (RM) is a rare but serious complication of rheumatoid arthritis.

RM may not correlate with disease activity nor disease duration.

Diagnosis is a challenge due to its wide clinical phenotype as well as the non-specificity of imaging, lumbar puncture and serological findings.

Treatment options are based on case reports and series and include corticosteroids, rituximab and cyclophosphamide.

